# Supplementary material for: Profiling the Targets of Protective CD8+ T Cell Responses to Infection
Source: Mol Ther Methods Clin Dev. 2017 Aug 18;7:20–31. doi: 10.1016/j.omtm.2017.08.003 (PMC5602877; doi:10.1016/j.omtm.2017.08.003)
Supplement: Document S1. Tables S1 and S2 [file mmc1.pdf]

OMTM, Volume 7

Supplemental Information

Profiling the Targets of Protective CD8$^+$ T Cell Responses to Infection

Joseph T. Bruder, Ping Chen, Greg Ekberg, Emily C. Smith, Christopher A. Lazarski, Bennett A. Myers, Jessica Bolton, Martha Sedegah, Eileen Villasante, Thomas L. Richie, C. Richter King, Joao C. Aguiar, Denise L. Doolan, and Douglas E. Brough

Supplemental Information:

Table S1. Vector yields on various size plates

Plate Size	Adg.*Py*HEP17		Adg.CMVp65	
	ffu/ml	ffu total	ffu/ml	ffu total
60 mm	3.10E+08	1.30E+09	8.20E+08	3.44E+09
6 well	4.55E+08	9.55E+08	6.70E+08	1.41E+09
12 well	9.00E+08	7.56E+08	1.10E+09	9.24E+08
24 well	1.33E+09	5.59E+08	1.02E+09	4.28E+08
48 well	1.38E+09	5.80E+07	1.27E+09	5.30E+07
96 well	1.26E+09	2.26E+07	1.19E+09	2.10E+07

Table S2. Thirteen antigens that recalled $CD8^+$ T cell responses more frequent than CSP from mice immunized with *P. yoelii* SPZ+CQ

Rank	Gene ID *P. yoelii*	Gene ID *P. falciparum*	% Identity [a]	*P. yoelii* Protein Name - Function	Liver stage active genes from Tarum et al., 2008	
					Transcriptome Clusters [h]	Proteome
1	PY02605	PF3D7_0932900	40	Hypothetical Protein	C3	-
2	PY02989	PF3D7_0212800	47	Hypothetical Protein [b]	C4	-
3	PY02326	PF3D7_1309500	74	snornp protein gar1 [c]	C3	+
4	PY02030	PF3D7_0624000	86	hexokinase		+
5	PY00629	PF3D7_0108300	33	asparagine-rich protein, putative		+
6	PY07062	PF3D7_1446400	82	pyruvate dehydrogenase E1 beta subunit	C3	+
7	PY00361	PF3D7_1357100	99	translation elongation factor EF-1, subunit alpha		+
8	PY04421	PF3D7_1121600	56	U43539 hepatocyte erythrocyte protein 17 kDa [d]	C3	+
9	PY05048	PF3D7_1108400	95	Protein kinase domain [e]		+
10	PY07825	PF3D7_0814200	86	Arabidopsis thaliana At1g20220/T20H2_3-related [f]	C3	+
11	PY05748	PF3D7_0930300	32	merozoite surface protein 1 precursor	C1	-
12	PY04475	PF3D7_0621200	86	ethylene-inducible protein hever [g]		+
13	PY02148	PF3D7_1334600	31	merozoite surface protein 7 precursor	C1	-

[a] Percent identity between *P. falciparum* and *P. yoelii* orthologues established using blastpp suite-2sequences, National Center for Biotechnology Information.

[b] *P. falciparum* protein function is multidrug efflux pump, putative.

[c] *P. falciparum* protein function is H/ACA ribonucleoprotein complex subunit 1, putative.

[d] *P. falciparum* protein function is exported protein 1.

[e] *P. falciparum* protein function is casein kinase 2, alpha subunit.

[f] *P. falciparum* protein function is DNA/RNA-binding protein Alba 1.

[g] *P. falciparum* protein function pyridoxine biosynthesis protein PDX1.

[h] Clusters from Tarum *et al*., 2008 - C1 includes LS genes mainly up-regulated in LS24 compared with LS40 and LS50, but that were also expressed in the mosquito and erythrocytic stages.C2 encompassed genes that were mainly up-regulated at LS24; C3 showed most of the genes up-regulated in all three LS time points queried. C4 showed genes mainly up-regulated in LS40 and LS50.
