# Supplementary material for: Profiling the Targets of Protective CD8+ T Cell Responses to Infection
Source: Mol Ther Methods Clin Dev. 2017 Aug 18;7:20–31. doi: 10.1016/j.omtm.2017.08.003 (PMC5602877; doi:10.1016/j.omtm.2017.08.003)
Supplement: Document S2. Article plus Supplemental Information [file mmc2.pdf]

Molecular Therapy
Methods & Clinical Development
Original Article

Profiling the Targets of Protective CD8$^+$ T Cell Responses to Infection

Joseph T. Bruder,[1,6] Ping Chen,[1] Greg Ekberg,[1] Emily C. Smith,[2,3] Christopher A. Lazarski,[1] Bennett A. Myers,[1] Jessica Bolton,[2,3] Martha Sedegah,[2] Eileen Villasante,[2] Thomas L. Richie,[2] C. Richter King,[1,7] Joao C. Aguiar,[2,4] Denise L. Doolan,[5] and Douglas E. Brough[1]

[1]GenVec, Inc., 910 Clopper Road, Suite 220N, Gaithersburg, MD 20878, USA; [2]Malaria Department, Naval Medical Research Center (NMRC), 503 Robert Grant Avenue, Silver Spring, MD 20910, USA; [3]Henry M. Jackson Foundation for the Advancement of Military Medicine, Inc., 6720A Rockledge Drive, Suite 100, Bethesda, MD 20817, USA; [4]Camris International, 3 Bethesda Metro Center, 16th Floor, Bethesda, MD 20814, USA; [5]Australian Institute of Tropical Health and Medicine, James Cook University, McGregor Road, Cairns, QLD 4870, Australia

T cells are critical effectors of host immunity that target intracellular pathogens, such as the causative agents of HIV, tuberculosis, and malaria. The development of vaccines that induce effective cell-mediated immunity against such pathogens has proved challenging; for tuberculosis and malaria, many of the antigens targeted by protective T cells are not known. Here, we report a novel approach for screening large numbers of antigens as potential targets of T cells. Malaria provides an excellent model to test this antigen discovery platform because T cells are critical mediators of protection following immunization with live sporozoite vaccines and the specific antigen targets are unknown. We generated an adenovirus array by cloning 312 highly expressed pre-erythrocytic *Plasmodium yoelii* antigens into adenovirus vectors using high-throughput methodologies. The array was screened to identify antigen-specific CD8$^+$ T cells induced by a live sporozoite vaccine regimen known to provide high levels of sterile protection mediated by CD8$^+$ T cells. We identified 69 antigens that were targeted by CD8$^+$ T cells induced by this vaccine regimen. The antigen that recalled the highest frequency of CD8$^+$ T cells, PY02605, induced protective responses in mice, demonstrating proof of principle for this approach in identifying antigens for vaccine development.

INTRODUCTION

Almost all licensed vaccines are thought to mediate protection through antibody production; therefore, antigen discovery research and development has focused largely on the identification of antigens that induce protective antibodies.[1] The availability of serum, the ease of working with antibodies, and, more recently, advances in microarray technology have facilitated these efforts. However, vaccine development for some of the most devastating infectious diseases, such as malaria, tuberculosis (TB), and HIV, has met with limited success, partially because these organisms have intracellular life cycle stages that are not targeted by antibodies, and they have developed sophisticated mechanisms to avoid clearance by host immune responses.[2] Since T cells have been implicated in protection from these diseases,[3–7] considerable efforts have been directed at developing vaccines that induce protective T cell responses. However, for infectious agents with large genomes that express many potential T cell antigens such as parasites and bacteria, many of the specific antigens that are targeted by protective CD8$^+$ T cells are not known. Identification of the target antigens of protective T cell responses would greatly facilitate vaccine development.

Malaria killed approximately 429,000 people in 2015,[8] most of them children, in sub-Saharan Africa. Despite decades of effort, a highly effective malaria vaccine is not available. Immunization with attenuated *Plasmodium* sporozoites can provide high levels of protection in mice, non-human primates, and humans.[9–12] Protection is mediated by CD8$^+$ T cells, which target a set of mostly unknown pre-erythrocytic stage antigens.[13–18] Activated CD8$^+$ T cells can kill infected hepatocytes, thereby preventing blood-stage infection, which is responsible for the clinical symptoms of the disease. However, substantial delivery issues are a considerable barrier to licensure of live sporozoite-based vaccines, and broad protection against circulating strains has not been demonstrated. An alternative approach is to identify the targets of these protective CD8$^+$ T cell responses and formulate them into a multivalent subunit vaccine designed to induce sustained T cell immunity.

The two *P. falciparum* sporozoite vaccines that are associated with high levels of protection in humans are radiation-attenuated sporozoites (RAS) and live sporozoites with concomitant chloroquine treatment to kill newly emerging blood-stage parasites (SPZ+CQ).

Received 10 August 2017; accepted 14 August 2017;
http://dx.doi.org/10.1016/j.omtm.2017.08.003.

[6]Present Address: Summit Consulting, 567 Chestertown Street, Gaithersburg, MD 20878, USA

[7]Present Address: PATH's Malaria Vaccine Initiative, 455 Massachusetts Avenue NW, Suite 1000, Washington, DC 20001, USA

Correspondence: Joseph T. Bruder, Summit Consulting, 567 Chestertown Street, Gaithersburg, MD 20878, USA.
E-mail: joetbruder@gmail.com

 © 2017 GenVec, Inc.
This is an open access article under the CC BY license (http://creativecommons.org/licenses/by/4.0/).

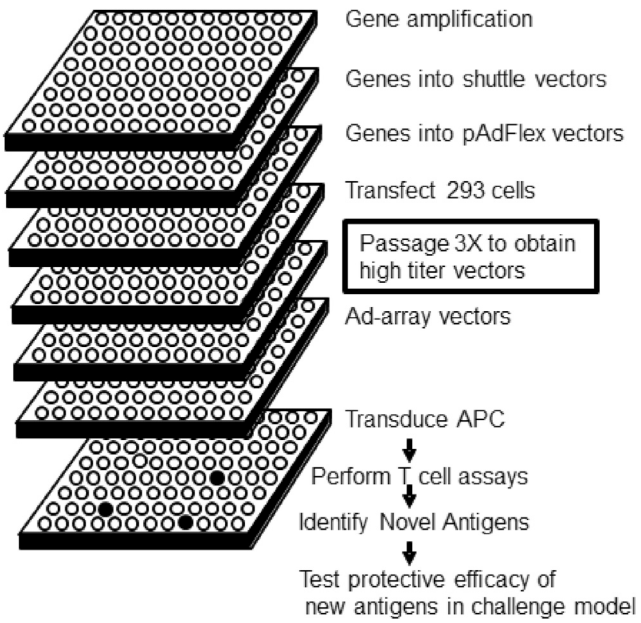

Figure 1. Schematic View of High-Throughput Ad-Array Generation and Antigen Identification Assays

The general steps involved in generating a defined array of adenovectors and their use in antigen discovery screens using high-throughput technology are indicated.

Immunization with RAS leads to infection of hepatocytes and expression of a set of early liver-stage genes, but these attenuated sporozoites do not develop into late liver and blood stages.[19] In BALB/c mice, the protective T cell response following vaccination with RAS is dominated by CD8$^+$ T cells specific for the major surface protein on the sporozoite, the circumsporozoite protein (CSP), although T cell responses specific for other antigens can also contribute to protection.[20] In humans, T cell responses specific for several antigens have been observed following RAS immunization.[21–23] In contrast to RAS, vaccination with SPZ+CQ allows expression of the full repertoire of liver-stage genes and replication of the parasite in hepatocytes.[24] Unlike RAS, where protection requires approximately 1,000 bites from infected mosquitoes, SPZ+CQ can provide durable protection in volunteers with as few as 30–45 bites.[25] This robust protection is strictly dependent on CD8$^+$ T cells[26] and immune response to CSP is not required, highlighting the fact that the specific antigen targets of protective immunity are not known.[27]

In this report, we describe a novel platform for the discovery of antigens that are the targets of T cell responses to infection (Figure 1). Using this system, we identified 69 pre-erythrocytic antigens that were targeted by CD8$^+$ T cell responses in mice immunized with protective regimens of *P. yoelii* SPZ+CQ. Moreover, we demonstrated that the antigen that recalled the highest frequency of interferon gamma (IFNγ)-expressing CD8$^+$ T cells, PY02605, provided sterile protection in mice when delivered in a DNA prime-adenovector boost regimen.

RESULTS

Generation of an Array of Adenovectors That Express a Panel of Highly Expressed *P. yoelii* Pre-erythrocytic Antigens

Pre-erythrocytic antigens, which are expressed in the sporozoite and liver stages of the *Plasmodium* spp. life cycle, are particularly promising targets for malaria vaccine development, with great potential to prevent infection and transmission.[28] The pre-erythrocytic stages of the parasitic life cycle are vulnerable to vaccine intervention because their antigens are expressed at a time when low numbers of sporozoites are transmitted by the mosquito to the human host and only a few hepatocytes become infected. We selected *P. yoelii* pre-erythrocytic genes with identifiable *P. falciparum* orthologs for generation of an adenovector array (Ad-array) based on their level of expression in microarray[29–31] and protein mass spectrometry[29] datasets. Gene selection was made without regard to protein function or subcellular localization. In total, 312 *P. yoelii* genes were amplified from genomic DNA and cloned into E1/E3-deleted adenovirus type 5 (Ad5) vector genomes (Figure 2).

To facilitate high-throughput production of the Ad-array, we compared the efficiency of adenovector generation in multi-well plates of different sizes. The adenovector plasmid had to convert into an adenovirus vector in sufficient quantities and quality to function in the antigen screening assay. Initially, we tested conversions of two pAdFlex plasmids that expressed the *P. yoelii* Hep17 antigen (AdgHep17) and the cytomegalovirus p65 antigen (AdgCMVp65). These large plasmids were transfected into 293 cells in 60-mm, 6-well, 12-well, 24-well, 48-well, and 96-well plates, and the cells were passaged to increase the adenovector titer. We observed efficient adenovector conversion in all of the wells as indicated by full cytopathic effect (CPE) at passage 2. Vector identity was verified by PCR using oligonucleotides that spanned the expression cassette (Figure 2B). Vector titers from each of the CPE wells (Table S1) demonstrated equivalent yields per infected cell. These results indicated that multiple adenovectors can be generated from pAdFlex adenovector plasmids in a parallel process in multi-well plates and that 96-well plates were suitable for the generation of the Ad-array.

The Screening System

The overall design of our antigen screening system is shown in Figure 3A. To test the elements of the screen, we first determined the MOI necessary to efficiently infect A20 cells. Cells were infected with various doses of AdGFP, an Ad5 vector expressing GFP, and the percentage of infected cells was measured 48 hr post-infection (Figure 3B). MOIs of 10, 100, or 1,000 focal forming units (ffus)/cell were required to infect approximately 2%, 10%, or 50% of the cells, respectively. To determine if adenovirus vectors could efficiently present antigen following infection of antigen presenting cells (APCs), we immunized BALB/c mice with a *Py*CSP-expressing plasmid, stimulated splenocytes from these mice with APCs infected with an Ad5 vector expressing *Py*CSP (Ad*Py*CSP), and measured activated T cells by the enzyme linked immunosorbent spot (ELISpot) assay. We observed strong recall responses to the Ad*Py*CSP-infected

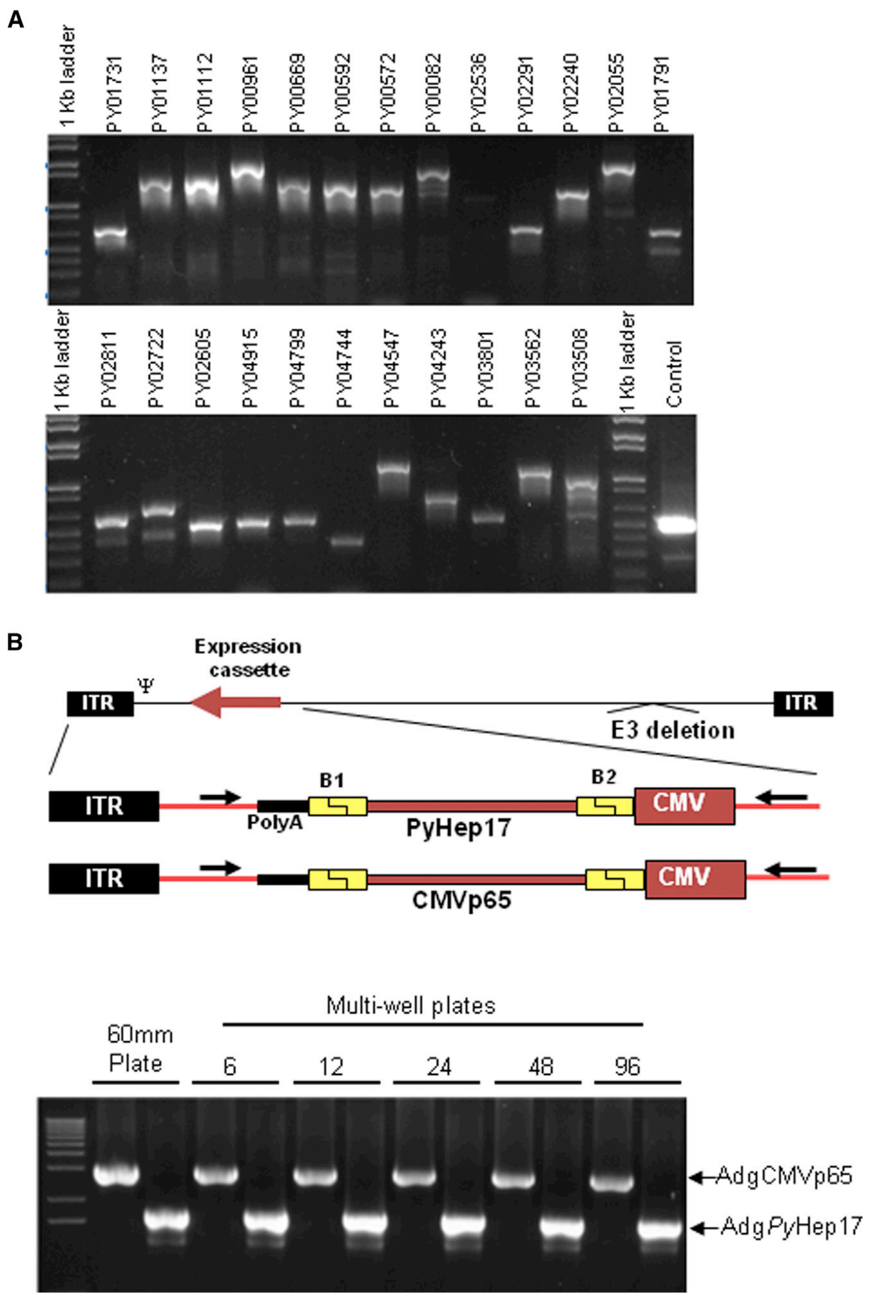

Figure 2. Generating the Ad-Array

(A) >300 *P. yoelii* pre-erythrocytic genes were amplified using *P. yoelii* genomic DNA and gene-specific primers. PCR products were electrophoresed on 1% agarose gels, showing a subset of 24 genes. The control is a pair of oligonucleotide primers used to amplify the E1 region of Ad5 DNA. (B) Parallel generation of two Ad-array vectors in multi-well plates. The schematic indicates two Ad-array vectors, Adg*Py*Hep17 and AdgCMVp65. The inverted terminal repeats (ITRs), CMV promoter, SV40 poly(A), and the 25-bp-long attB recombinase sequences that flank the transgene (B1 and B2) are indicated. Two plasmids, encoding *Py*Hep17 and CMVp65, were AdFlex linearized with Pac I and transfected into 293 cells in 60-mm, 6-well, 12-well, 24-well, 48-well, and 96-well plates. Following two passages in 293 cells in the same plate size, CPE was observed in all wells. Viral DNA was obtained, and PCR analysis was performed using primers that flank the expression cassette as indicated by the arrows in the schematic. The products of the PCR reaction were loaded into a 1% agarose gel and electrophoresed. Arrows next to Adg*Py*Hep17 and AdgCMVp65 indicate the expected size for the PCR products.

cells, even at a low MOI, comparable to those generated by pulsing APCs with a peptide containing the *Py*CSP immunodominant epitope (Figure 3C). Very low responses were seen in the negative controls. These results demonstrate that A20 cells (which express both major histocompatibility complex [MHC] class I and class II alleles) infected with Ad*Py*CSP are able to present antigen to immune T cells. This process was highly efficient, as strong T cell responses were observed even at an MOI of 10, a multiplicity that resulted in transduction of approximately 2% of the target cells. Increasing the MOI resulted in substantially increased A20 cell transduction (Figure 3B) but only marginally increased functional activity in the ELISpot assay (Figure 3C). Thus, low-level target cell transduction is sufficient for optimal activity to detect T cell responses in the ELISpot assay.

To determine whether lower-frequency T cell responses from mice immunized with sporozoite vaccines could be identified using our approach, we assayed $CD8^+$ T cell responses specific for *Py*CSP from mice immunized with protective regimens of RAS and SPZ+CQ. We chose *Py*CSP as the test antigen because it is the most well-characterized target of T cell responses from mice immunized with these regimens.[20,27] First, we assayed splenocytes from mice immunized with a highly protective three-dose regimen of RAS for the presence of *Py*CSP-specific T cells. We were able to recall *Py*CSP-specific T cells in splenocytes from these mice using Ad*Py*CSP-infected A20 cells in both ELISpot (Figure 4A) and intracellular cytokine staining (ICS) assays (Figure 4B). Low background responses were observed in the negative controls.

It was important to assess the degree of purity of the adenovector preparation necessary for the screen because if unpurified adenovectors were suitable, this would greatly simplify generation of the Ad-array. Accordingly, we compared highly purified Ad*Py*CSP (purified over three successive CsCl gradients) with cell lysates containing unpurified recombinant adenovector. *Py*CSP-specific $CD8^+$ T cell

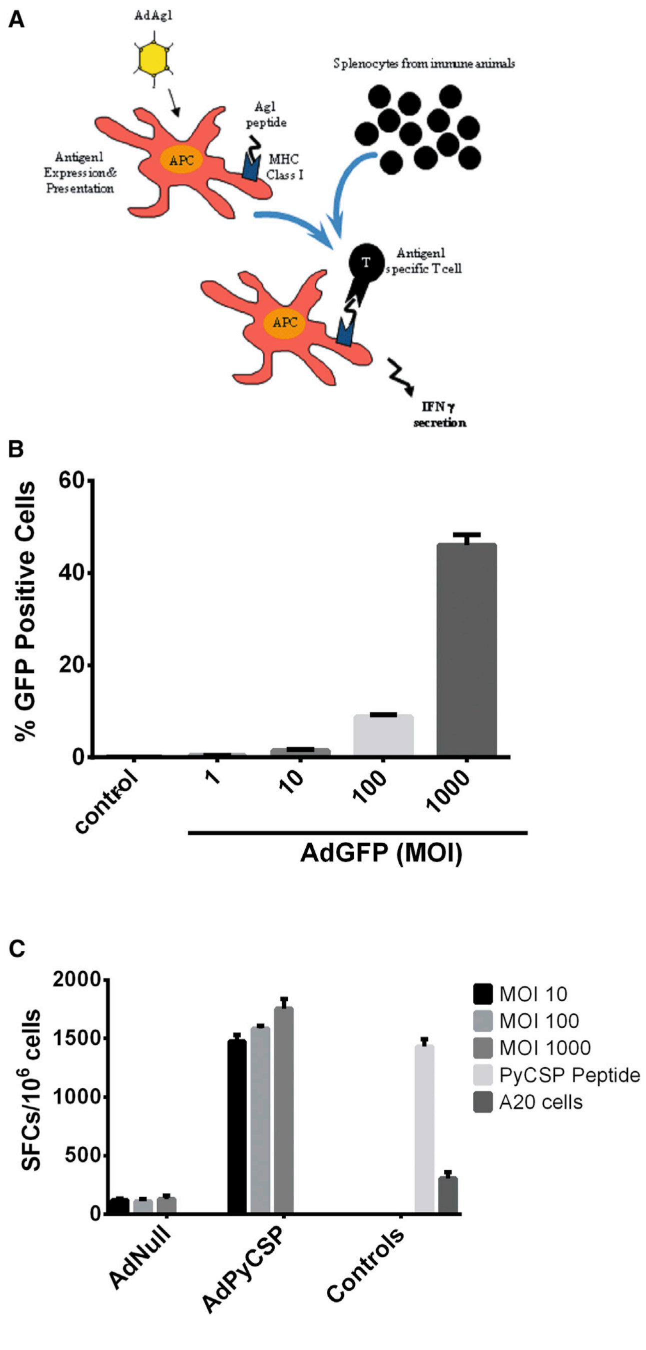

Figure 3. Adenovector-Expressed Antigen Is Effective at Recalling T Cell Responses from Immunized Mice

(A) Schema for in vitro antigen discovery. First, A20 APCs are infected with each adenovector in the array. After 24 hr, the specific antigen of interest is expressed and presented on MHC class I molecules. The infected APCs are incubated with splenocytes isolated from immune animals to identify CD8$^+$ T cells with specificity for the antigens expressed in the Ad-array. These antigen-specific CD8$^+$ T cells are identified by either ICS and flow cytometry or ELISpot assays measuring IFNγ secretion. (B) Ad5 vector effectively transduces APCs. A20 cells were infected with AdGFP at the indicated MOI. The percentage of GFP$^+$ cells was determined by flow cytometry. (C) Ad*Py*CSP-infected APCs can recall CD8$^+$ T cell responses from immune mice. Target A20 cells were infected with various MOIs of an Ad5 vector expressing *Py*CSP (Ad*Py*CSP). Control targets were uninfected A20 cells, A20 cells infected with various MOIs of an Ad5 vector that does not express a transgene (AdNull), and A20 cells stimulated with an immunodominant *Py*CSP peptide (amino acids 280–288, SYVPSAEQI). These targets were used to stimulate splenocytes from BALB/c mice immunized with a *Py*CSP DNA vaccine. IFNγ-expressing cells were measured by ELISpot. SFC (spot-forming cells); error bars indicate the SEM (n = 3). SFC, spot-forming cell.

responses were detected with both purified and unpurified vectors using ELISpot (Figure 4A) and ICS (Figure 4B) assays. Our results indicated that vector purification is not required to identify antigens that recall CD8$^+$ T cell responses in mice immunized with RAS.

Ad-array vectors contain 25bp-long attB sequences flanking the transgene (Figure 2B), which are remnants of the recombinase cloning reaction. We compared Ad-array vectors directly with our vaccine adenovectors, which do not carry the flanking attB sequences. Our results indicate that the attB sequences did not inhibit the capacity to recall T cell responses in mice (Figure 4C), indicating that Ad-array vectors are suitable for screening.

Mice immunized with a two-dose regimen of 200, 2,000, and 20,000 SPZ+CQ were completely protected from *P. yoelii* sporozoite challenge (Figure 4D). Figure 4E shows that *Py*CSP-specific T cells were induced by immunizing mice with a highly protective 2,000 SPZ+CQ regimen. Splenocytes from immunized mice had a high background of activated CD8$^+$ T cells. When incubated with A20 cells infected with the negative control vectors AdNull and AdGFP, 0.8%–0.9% of the CD8$^+$ T cells were activated. A20 cells infected with Ad*Py*CSP recalled *Py*CSP-specific T cell responses that were more frequent than the negative controls. Statistically significant results were observed with MOIs of 10 and 100 ffu/cell. These data suggested that it would be possible to utilize our Ad-array technology to identify new antigen targets of protective T cell responses following immunization of mice with SPZ+CQ.

Identification of the Antigen Targets of CD8$^+$ T Cells Induced following Vaccination with Protective Regimens of SPZ+CQ

To generate protective T cells for the identification of antigens, we used the 2,000 SPZ+CQ regimen and harvested splenocytes 2 weeks after the last sporozoite immunization. The full array was screened simultaneously, in triplicate, against these freshly isolated splenocytes by ICS to identify pre-erythrocytic stage antigens able to recall IFNγ-expressing CD8$^+$ T cells. A20 cells infected with 100 ffu/cell Adg*Py*CSP were included as a positive control. Negative controls included uninfected A20 cells and A20 cells infected with 100 ffu/cell of AdNull and AdGFP vectors. The mean of the negative controls was 1% IFNγ-expressing CD8$^+$ T cells (Figure 5). We defined antigens with responses greater than 2 SD of the mean of the negative controls (>1.2% CD8$^+$ IFNγ$^+$ cells) as positive hits in our screen. By this definition, 69 of the antigens in the array were positive and were targeted by CD8$^+$ T cells induced in mice immunized with SPZ+CQ (Figure 5; Table 1). Thirteen of these antigens recalled

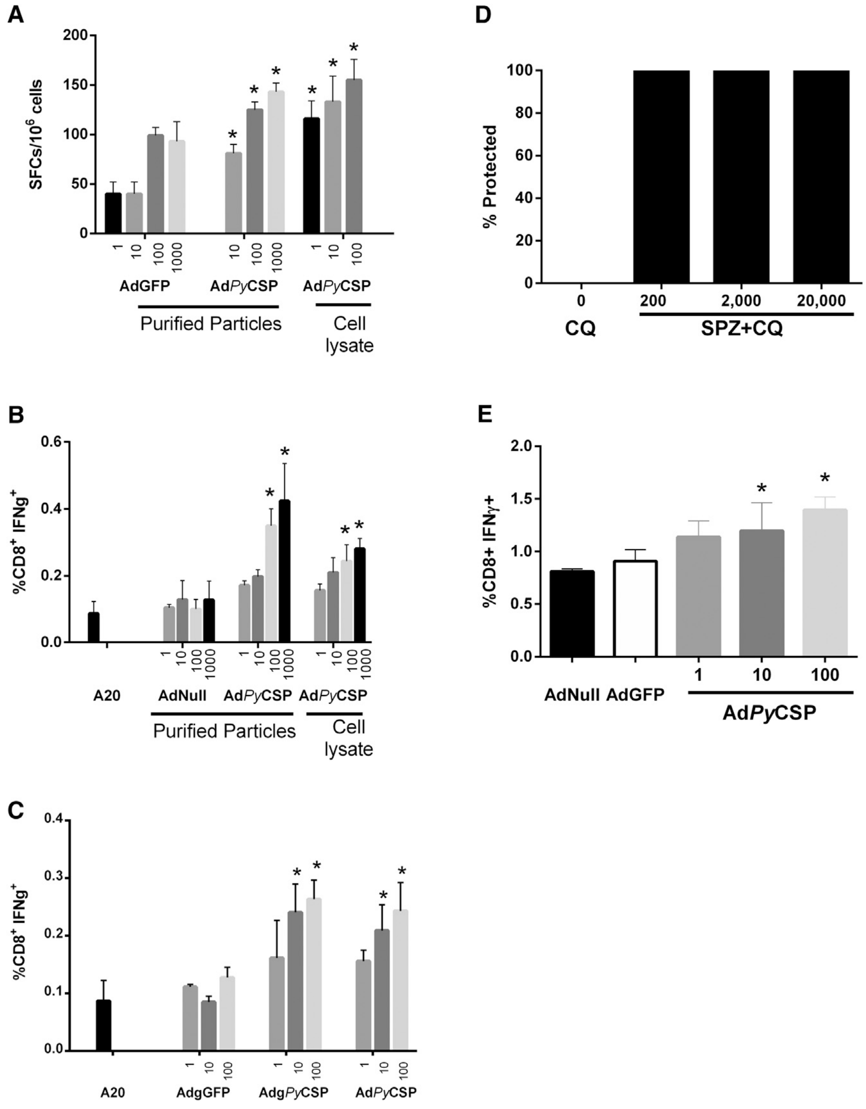

Figure 4. Adenovector-Expressed Antigen Is Effective at Recalling CD8⁺ T Cell Responses from Mice Immunized with Protective Regimens of Sporozoite Vaccines

Target A20 cells were infected at the indicated MOIs with Ad*Py*CSP (either triple $CsCl_2$ purified adenovector or unpurified cell lysate from adenovector infected cells) and incubated with splenocytes from RAS or SPZ+CQ-immunized mice. Control targets were A20 cells infected with AdNull, AdGFP, and uninfected A20 cells. (A) IFNγ-secreting cells from mice immunized with RAS were measured by ELISpot. (B) $CD8^+$ $IFN\gamma^+$ cells from mice immunized with RAS were measured by ICS and flow cytometry. (C) Comparison of Ad-array *Py*CSP vector (Adg*Py*CSP) with Ad*Py*CSP, which does not contain the recombination motifs flanking the expression cassette. $CD8^+$ $IFN\gamma^+$ cells from mice immunized with RAS were measured by ICS and flow cytometry. (D) Dose-response analysis for efficacy of SPZ+CQ vaccine regimen following immunization with 0, 200, 2,000, or 20,000 *P. yoelii* sporozoites with chloroquine treatment. Mice were assessed for sterile protection by blood smear. (E) Ad*Py*CSP-infected cells can recall $CD8^+$ T cell responses from mice immunized with 2,000 SPZ+CQ. Target A20 cells were infected with cell lysates containing the indicated Ad5 vectors and antigen-specific $CD8^+$ T cell responses were measured by ICS and flow cytometry. Error bars indicate the SEM (n = 3). Asterisks indicate statistically significant differences compared with AdGFP (A), A20 (B and C), or AdNull (E) controls ($p < 0.05$ by ANOVA with the Bonferroni mean-comparisons test).

higher-frequency $CD8^+$ T cell responses than *Py*CSP. The antigen that recalled the highest response was PY02605. We also analyzed $CD4^+$ T cell responses and tumor necrosis factor (TNF)-α and interleukin (IL)-2 cytokines by ICS. $CD4^+$ T cell responses were not observed in this system. $CD8^+$ TNF-α-expressing T cells were observed and tended to mirror the $CD8^+$ IFNγ responses. Very low levels of IL-2-expressing cells were observed (data not shown).

PY02605 Is a Protective Antigen

Since the SPZ+CQ regimen induces protective T cell responses directed against antigens expressed in the pre-erythrocytic stages of the parasite life cycle,[26,27,32] we hypothesized that a subset of antigens identified in the SPZ+CQ screen would induce protective immune responses when delivered using a potent vaccine regimen designed to optimize $CD8^+$ T cell responses. Accordingly, we compared antigens that ranked at the two extremes of our screening strategy, PY02605 and PY05837, for their capacity to protect mice against a sporozoite challenge. PY02605 recalled the highest frequency of $CD8^+$ T cells and PY05837 did not recall antigen-specific T cell responses in our screen. We tested the protective capacity of these antigens using a DNA prime-Ad5 boost regimen in BALB/c mice.[33] Mice were immunized with 100 μg of DNA vector expressing the specific antigen and then boosted 6 weeks later with 1×10^{10} particle units (PUs) of an Ad5 vector expressing the same antigen. Two weeks after the Ad5 boost, mice were challenged with *P. yoelii* sporozoites and protection was monitored by microscopic examination of Giemsa-stained blood smears. 43% of the PY02605 immunized mice were sterilely protected (6 of 14 mice), indicating that PY02605 can provide protection in mice (Table 2). Only 1 mouse from the group of 14 immunized with the PY05837 antigen was negative for blood-stage parasitemia following challenge, suggesting that this antigen is not protective. The positive control group, which was immunized with *Py*CSP-expressing DNA and Ad5 vectors, protected 100% of the mice. The negative control group, immunized with DNA and Ad5 vectors that did not express any transgene (Null) did not protect any mice (Table 2). These data indicate that our antigen discovery system is capable of identifying protective antigens.

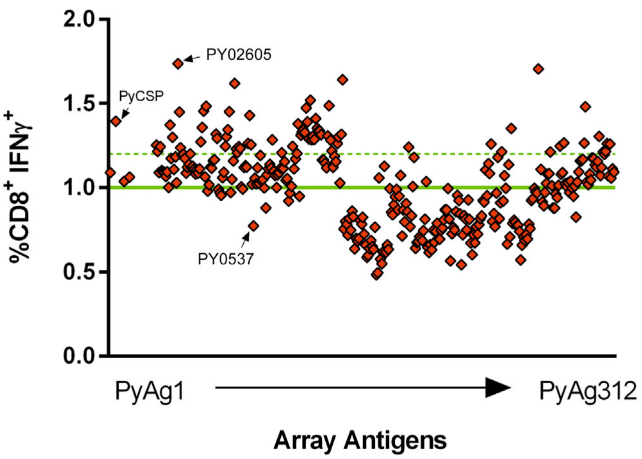

Figure 5. Identification of Targets of CD8+ T Cell Responses Induced by a Highly Protective SPZ+CQ Vaccine Regimen

Splenocytes from BALB/c mice immunized with SPZ+CQ were screened for CD8+ recall responses stimulated by 312 pre-erythrocytic antigens and evaluated by ICS and flow cytometry. The mean of the negative controls is indicated by the horizontal green line. The dotted green line indicates responses that are ≥2 SD above the mean of the negative controls. Arrows indicate two antigens tested for protection shown in Table 2.

PF3D7_0932900 Is Immunogenic in Mice

To begin evaluation of a selected pre-erythrocytic antigen as a vaccine candidate, we cloned the *P. falciparum* ortholog of PY02605 into a highly immunogenic and low seroprevalent gorilla adenovector (GC46)[34,35] and tested immunogenicity in mice. PF3D7_0932900 was codon optimized for expression in mammals, synthesized, and used to produce GC46.PF3D7_0932900. We immunized BALB/c mice (n = 6/group) with a single intramuscular (IM) administration of GC46.PF3D7_0932900 (1×10^9 PU). GC46.Null immunized and naive mice were included as control groups. At 21 days post-immunization, mice were euthanized for T cell studies. Antigen-specific T cell responses were measured from splenocytes by flow cytometry after stimulation with overlapping peptide pools and staining for cytokines and cell surface markers (Figure 6). GC46.PF3D7_0932900 was highly immunogenic, inducing robust antigen-specific CD8+ and low-level CD4+ T cell responses.

DISCUSSION

Identification of CD8+ T cell targets of infectious disease has the potential to increase our understanding of cellular immunity and to facilitate the development of vaccines, diagnostics, and therapeutics. In this report, we describe a novel antigen discovery system and demonstrate its capacity to identify the antigen targets of protective T cell responses to infection. Although we demonstrated the feasibility of this system for malaria, we expect that the Ad-array technology and screens similar to the one employed here may also be useful for identifying the antigens targeted by T cell responses to other infectious agents or associated with other disease states. Arrayed adenoviral expression libraries have been produced previously and used to identify gene products involved in disease pathways[36]; however, to our knowledge, this work is the first demonstration of the use of Ad-array technology to identify the antigen targets of cellular immunity. We show that low titers of unpurified adenovirus vector, quantities that transduce only 2% of autologous APCs, are sufficient to recall antigen-specific T cell responses in mice immunized with highly protective regimens of live sporozoite vaccines. Our ability to use low quantities of unpurified vector in the antigen discovery screen simplifies high-throughput Ad-array production and indicates potential for producing large Ad-arrays encoding the full transcriptome of complex pathogens with large genomes.

Advantages of the Ad-array technology over other T cell antigen discovery efforts are that adenoviruses efficiently infect many cell types including APCs[37] and that, following infection, full-length proteins are expressed and multiple undefined epitopes are presented, similar to what occurs during a natural infection. The Ad-array technology was as effective as the immunodominant *Py*CSP peptide in detecting *Py*CSP-specific CD8+ T cell responses. Moreover, this technology was highly sensitive, detecting low-level *Py*CSP-specific CD8+ T cell responses following immunization with RAS and SPZ+CQ. Other T cell antigen discovery efforts utilized in silico algorithms to predict epitopes with high MHC binding affinity and then generated libraries of peptides corresponding to the selected epitopes.[21–23,38–41] Although these methods are improving, they are expensive and limited in their capacity to predict protective T cell epitopes.[42,43] In the mouse malaria models, epitope prediction algorithms have supported the identification of several pre-erythrocytic antigens that are targeted by T cell responses induced following immunization with *P. yoelii* and *P. berghei* RAS or SPZ+CQ; however, these antigens have not demonstrated protective efficacy when tested in challenge experiments.[38–40]

We focused on identifying T cell responses specific for pre-erythrocytic antigens following immunization with a highly protective SPZ+CQ vaccine regimen because of the potential for increased diversity of antigen responses associated with this regimen relative to RAS.[44,45] Using this regimen, we identified a set of 69 pre-erythrocytic stage antigens that were targeted by vaccine-induced CD8+ T cells. Thirteen of these antigens recalled more robust responses than *Py*CSP and were expressed in the liver stage of the *P. yoelii* life cycle (Table S2).[29] The antigen that recalled the most frequent CD8+ T cell responses, PY02065, is a 173 amino acid conserved *Plasmodium* protein with unknown function that is upregulated throughout the liver stage of the *P. yoelii* life cycle.[29] Since protection induced by the SPZ+CQ regimen is dependent on CD8+ T cells, we hypothesized that a subset of the 69 antigen-specific CD8+ T cells that we identified may contribute to the robust protection observed with this regimen. We tested this hypothesis by evaluating the protective efficacy induced by a DNA prime-Ad5 boost regimen expressing the antigen that recalled the most frequent CD8+ T cells, PY02065. This robust T cell vaccine regimen was partially protective, inducing sterile protection (absence of blood-stage infection) in 6 of 14 mice following *P. yoelii* sporozoite challenge. This finding strongly suggests that our antigen discovery system is effective at identifying the targets

Table 1. *P. yoelii* Genes that Recalled T Cell Responses >2 SDs Higher than the Mean of the Negative Controls

SPZ+CQ Rank	*P. yoelii* Gene ID	$CD8^+$ $IFN\gamma^+$ (%)	Size (nt)	GenVec ID	SPZ+CQ Rank	*P. yoelii* Gene ID	$CD8^+$ $IFN\gamma^+$ (%)	Size (nt)	GenVec ID
1	PY02605	1.74	522	GV0014	36	PY02240	1.30	1,131	GV0011
2	PY02989	1.71	1,292	GV0350	37	PY02076	1.29	1,095	GV0041
3	PY02326	1.64	624	GV0139	38	PY00463	1.29	972	GV0112
4	PY02030	1.62	1,485	GV0054	39	PY01621	1.29	2,958	GV0117
5	PY00629	1.52	2,877	GV0113	40	PY00808	1.28	1,359	GV0114
6	PY07062	1.49	939	GV0129	41	PY05019	1.28	1,134	GV0084
7	PY00361	1.48	1,332	GV0033	42	PY00142	1.28	1,950	GV0130
8	PY04421	1.48	390	GV0402	43	PY06454	1.27	525	GV0030
9	PY05048	1.47	1,008	GV0110	44	PY04001	1.26	444	GV0372
10	PY07825	1.46	726	GV0032	45	PY02435	1.26	408	GV0401
11	PY05748	1.45	5,319	GV0050	46	PY01556	1.26	1,836	GV0135
12	PY04475	1.43	894	GV0066	47	PY03629	1.26	1,509	GV0065
13	PY02148	1.41	972	GV0118	48	PY05566	1.26	1,116	GV0446
14	PY03168	1.39	1,194	CSP	49	PY04605	1.26	645	GV0294
15	PY05088	1.39	630	GV0111	50	PY05369	1.26	1,308	GV0451
16	PY06246	1.38	1,209	GV0104	51	PY01620	1.26	2,151	GV0067
17	PY01731	1.37	708	GV0009	52	PY00082	1.25	1,716	GV0001
18	PY07531	1.36	438	GV0031	53	PY03102	1.25	360	GV0369
19	PY02175	1.35	1,377	GV0119	54	PY04293	1.25	1,086	GV0099
20	PY03289	1.35	894	GV0326	55	PY04306	1.24	633	GV0046
21	PY01848	1.34	744	GV0107	56	PY03138	1.24	1,680	GV0058
22	PY04028	1.34	1,398	GV0049	57	PY00592	1.24	1,410	GV0003
23	PY03601	1.34	1,524	GV0122	58	PY02214	1.24	525	GV0196
24	PY03098	1.34	2,058	GV0121	59	PY02722	1.24	705	GV0015
25	PY01182	1.33	1,716	GV0106	60	PY00920	1.23	1,239	GV0059
26	PY01906	1.33	2,643	GV0108	61	PY03879	1.23	1,875	GV0055
27	PY00858	1.32	1,419	GV0040	62	PY00382	1.22	633	GV0131
28	PY02102	1.32	696	GV0138	63	PY00723	1.21	936	GV0439
29	PY04079	1.31	828	GV0123	64	PY00572	1.21	1,326	GV0002
30	PY00935	1.31	1,506	GV0105	65	PY01758	1.21	825	GV0320
31	PY06529	1.31	1,847	GV0128	66	PY07442	1.21	852	GV0436
32	PY01558	1.31	2,547	GV0116	67	PY03674	1.21	1,686	GV0360
33	PY00196	1.30	738	GV0431	68	PY03081	1.21	3,657	GV0057
34	PY03814	1.30	3,690	GV0048	69	PY03150	1.21	891	GV0092
35	PY01863	1.30	954	GV0137					

nt, nucleotide.

of protective T cell responses to malarial infection, although additional antigens that recalled $CD8^+$ T cells in our screen will need to be tested to further confirm our hypothesis.

Our malaria vaccine development efforts focus on improving DNA-Ad5-CA, a first-generation vaccine candidate that induced an encouraging 27% sterile protection in human volunteers challenged with controlled human malaria infection (CHMI).[40] DNA-Ad5-CA is a DNA prime-Ad5 boost regimen that expresses two *P. falciparum* antigens: *Pf*CSP and apical membrane antigen 1 (*Pf*AMA1). $CD8^+$ T cell responses specific for *Pf*AMA1 were associated with protection, and 2 of the 4 protected volunteers had the highest frequency of $CD8^+$ T cell responses specific for *Pf*CSP. Our goal is to increase vaccine efficacy to meet the preferred product characteristic target of 75% efficacy set forth by the World Health Organization (WHO).[46] Since overall efficacy of DNA-Ad5-CA was

Table 2. Immunization with PY02605 Partially Protects BALB/c Mice from *P. yoelii* Sporozoite Challenge

Vaccine Antigen	Protected/Total	Protection (%)	p Value[a]
Null	0/14	0	NA
PyCSP	14/14	100	<0.001
PY02605	6/14	43	0.016
PY05837	1/14	7	1

[a]Fisher's exact test (two-sided) versus null. Alpha < 0.05.

limited by pre-existing immunity to the Ad5 vector,[40] we have selected a highly immunogenic gorilla-based adenovector, GC46,[34,35,47] for advancement and have recently demonstrated superior immunogenicity and protection with this vector relative to Ad5 in a malaria model.[47] In animal models, GC46 performance is similar to chimpanzee adenovirus type 3,[48] and given the excellent safety, immunogenicity, and efficacy data that have been achieved with the chimpanzee-based adenovirus vectors in humans,[49–54] we are enthusiastic about the potential to test GC46 vectors expressing CSP and AMA-1 in the clinic. Additional improvements in efficacy can be obtained by increasing the number of antigens in this vaccine regimen.[55,56] In this regard, PF3D7_0932900, the *P. falciparum* ortholog of PY02065, and the *P. falciparum* orthologs of other antigens identified in this work have potential to increase the efficacy of the DNA-Ad5-CA vaccine candidate.

MATERIALS AND METHODS

Primer Design, Gene Amplification, and Plasmid Cloning

Highly abundant *P. yoelii* pre-erythrocytic antigens with identifiable *P. falciparum* orthologs were selected for inclusion in the Ad-array. We included genes encoding proteins ≥130 amino acids in length because larger proteins have a greater probability of containing T cell epitopes. The DNA sequences of the selected genes used for amplification were obtained from the PlasmoDB database.[57] All of the single exon genes were amplified directly from *P. yoelii* (17XNL) genomic DNA using a pair of gel purified primers: the forward (fwd) primer contains the Kozak consensus CACC upstream of the starting codon ATG, the reverse (rev) primer retains the native stop codon at the end of the oligonucleotide, and both of the primers were designed to have the same melting temperature (Tm) at 66°C. PCR amplification was performed using high-fidelity platinum Taq polymerase (Invitrogen, Carlsbad, CA) or Hot Star polymerase (QIAGEN, Gaithersburg, MD). For multi-exon genes, we amplified individual exons first, and then assembled individual exons into an intact gene product using the cDNA synthesis PCR.[58] All PCR-amplified gene products were cloned into a shuttle vector, pCR8/GW/TOPO (Invitrogen, Carlsbad, CA). The correct orientation of the clones was confirmed by DNA sequencing using M13 fwd and M13 rev oligonucleotides. The pAdFlex plasmids pACE1(3511rfC)E3(10X) and pACE1(t.rfC.MCS)E3(10X) contain the full-length Ad5 genome with deletions in the E1 and E3 regions. Within the E1 region, we inserted *att*B1 and *att*B2 elements for lambda recombination and a CMV promoter and poly(A) sequences for antigen expression. The genes in the shuttle vector were moved into these destination gateway-converted adeno plasmids with high efficiency using the Gateway LR reaction[59] (Invitrogen, Carlsbad, CA), in 96-well plate format to generate an array of pAdFlex plasmids expressing pre-erythrocytic genes from *P. yoelii* (pAdg*Py*1-360).

Ad-Array Viral Vector Construction

The Ad-array is an array of adenovirus vectors expressing *P. yoelii* antigen genes constructed from an array of pAdFlex plasmids. Briefly, the recombinant Ad5 genomes containing an antigen gene expression cassette in the E1 region were liberated from pAdFlex plasmids by digestion with Pac I or I-CeuI restriction endonuclease, which cleaves adjacent to the inverted terminal repeats (ITRs) of the Ad5 genome. This vector DNA (60 ng) was transfected into monolayer 293 cells seeded at 70,000 cells per well in 96-well plates using 0.7 μL polyfect transfection reagent (QIAGEN, Gaithersburg, MD). Three days later, transfected cells were frozen and thawed three times to lyse the cells, releasing infectious recombinant virus, and 18 μL of these lysates was used to infect fresh 293 cells in another 96-well plate. Cell lysates were serially passaged in this manner every 3 days until CPE was observed. The identity of the adenovector was determined using primers Ad5s278 (CGCGGGAAAACTGAATAAGA) and Ad5a3598 (GCTGCTGCAAAACAGATACA) as indicated in Figure 2B. Vector yields were determined by using the ffu assay[60] on a subset of approximately 10% of the array to ensure that sufficient quantities of vector were being produced.

Production of Adenovirus Vectors for Protection Studies in Mice

To generate sufficient vector for mouse protection experiments, residual CPE lysates were expanded in four 10-cm plates and the cell lysates were harvested 3 days later. The expanded cell lysates were analyzed to determine infectious vector particle concentration, were analyzed by PCR to ensure vector genomic integrity, and were used to seed a production run in suspension 293-ORF6 cells in shaker flasks using serum-free media. Three days after infection, the recombinant vectors were released from infected cells by three cycles of freeze-thawing, treated with benzonase (EMD Millipore, Billerica, MA), purified by banding on two successive CsCl gradients, dialyzed into final formulation buffer and stored at −80°C. Physical PUs were determined by absorbance at 260 nm following disruption of the capsid with SDS.

Mice, Parasites, and Cells

The study protocols were reviewed and approved by the Walter Reed Army Institute of Research/Naval Medical Research Center (NMRC) and GenVec Institutional Animal Care and Use Committees in compliance with all applicable federal regulations governing the protection of animals in research. Female (6- to 8-week-old) BALB/c mice were purchased from Harlan Laboratories (http://www.envigo.com) or Charles River Laboratories (Wilmington, MA). CD1 outbred mice (5–6 weeks old) were purchased from Charles River Laboratories (Wilmington, MA). *P. yoelii* (17XNL non-lethal strain, clone 1.1) parasites were maintained by alternating passage in *Anopheles stephensi* mosquitoes and female CD1 mice. HEK293 cells, a

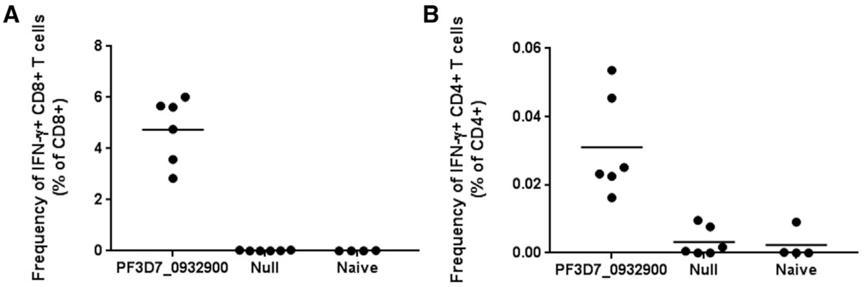

Figure 6. The *P. falciparum* Ortholog of PY02605, PF3D7_0932900, Is Immunogenic in BALB/c Mice

Mice were immunized with 1×10^9 PU of GC46.PF3D7_0932900 or a control adenovector that does not express a transgene, GC46.Null. Three weeks post-immunization, mice were euthanized and PF3D7_0932900-specific $CD8^+$ (A) and $CD4^+$ (B) T cell responses were measured by ICS and flow cytometry following 4-hr stimulation using pooled overlapping 15-mer peptides.

human embryonic kidney cell line transformed by sheared adenovirus type 5 DNA, were obtained from the American Type Culture Collection (Rockville, MD, USA) and maintained in DMEM supplemented with 10% calf serum. The A20.2J (ATCC clone HB-98) B cell line derived from BALB/c mice, which expresses both class I and class II MHC genes,[61] was purchased from ATCC and maintained in RPMI-1640 medium supplemented with 20% fetal bovine serum (FBS) and 1% glutamine.

Immunization Regimens

RAS were generated by exposing salivary gland *P. yoelii* sporozoites to 10,000 rads. To obtain splenocytes for testing recall responses to *Py*CSP, female BALB/c mice were immunized, via tail vein injection, with three doses of RAS (10,000, 5,000, and 5,000) at 3-week intervals.

For SPZ+CQ immunizations, female BALB/c mice were immunized with two administrations (1 month apart) of live infectious *P. yoelii* sporozoites. Various doses of sporozoites were tested (group 1 = 20,000, group 2 = 2,000, group 3 = 200, group 4 = 0). Infected mice received a 0.1mL intraperitoneal injection of a solution of chloroquine hydrochloride (Sigma-Aldrich, St. Louis, MO) 8 mg/mL diluted in PBS, to kill newly emerging blood-stage parasites, starting on the same day as sporozoite immunizations and continuing for 10 consecutive days following each sporozoite immunization. For both SPZ+CQ and RAS immunizations, most of these mice were euthanized 2 weeks after the last dose to obtain splenocytes for T cell analysis. However, a subset of the mice was infected with *P. yoelii* sporozoites to insure that 100% sterile protection had been achieved, as described below.

For plasmid DNA immunizations, BALB/c mice were immunized with 100 μg DNA vector, p*Py*CSP, in a 0.1 mL volume by intramuscular immunization as described previously.[62] Two weeks later, mice were euthanized to obtain splenocytes for analysis. For DNA-Ad5 immunizations, BALB/c mice were immunized with 100 μg of DNA vector, pcDNA3.2-Dest (Invitrogen, Carlsbad, CA) in a 0.1 mL volume by intramuscular immunization. Six weeks later, these mice were boosted with 1×10^{10} PU of Ad5 vector in a 0.1 mL volume. For both DNA and Ad5 administrations, we performed bilateral injections into the tibialis anterior muscles with a 0.3-mL syringe and a 29G1/2 needle (Becton Dickinson Co., Franklin Lakes, NJ).

For GC46.PF3D7_0932900 immunizations, BALB/c mice were immunized with a single dose of 1×10^9 PU of GC46.PF3D7_0932900 by the intramuscular route with a 1-mL syringe and a 30G needle (Becton Dickinson Co., Franklin Lakes, NJ). At 21 days post-immunization, mice were euthanized for splenocyte harvest and assessment of immune responses by ICS and flow cytometry.

Protection Studies

Protection studies were performed as previously described.[55] Mice were challenged intravenously in the tail vein with 200 *P. yoelii* sporozoites using a 1-mL syringe and 26G1/2 needle. Sporozoites were hand dissected from infected mosquito salivary glands[63] and diluted for challenge in M199 medium containing 5% normal mouse serum (Gemini Bio-Products, West Sacramento, CA). The development of parasitemia was monitored over the next 2 weeks by microscopic examination of Giemsa-stained blood smears. Mice were considered protected if no parasites were observed in any sample at day 6, day 9, or day 14 post-challenge.

Infection of A20 Cells

A20.J2 cells (ATCC, Manassas, VA), also referred to as A20 cells, are a B cell line derived from BALB/c mice that expresses both class I and class II major histocompatability complex genes. A20 cells were grown in 15 mL fresh RPMI-1640 media plus 20% FBS and 1% L-glutamine in 25-mL T-flasks. The T-flasks were kept upright and incubated at 37°C in a 5% CO_2 incubator. When the cells reached a density of $1.2-1.8 \times 10^6$ cells/mL, they were used to seed 12-well plates at a density of 5.0×10^5 cells/well. The following day, the cells were infected with AdGFP, an adenovirus vector that expresses GFP, for 2 hr in a volume of 200 μL. After infection, cells were washed with PBS, overlaid with 1 mL fresh media, and incubated at 37°C and 5% CO_2 for 48 hr. The percentage of the GFP^+ cells was analyzed by flow cytometry. For the array screening, 6×10^5 A20.2J cells were infected with 150 μL CPE lysate from each of the Ad-array vectors in a volume of 350 μL in 24-well plates for 2 hr. After infection, 0.25 mL fresh media were added to each well and the infected cells were incubated at 37°C and 5% CO_2 for 24 hr before plating in the stimulations described below.

ICS and Flow Cytometry Analysis

Stimulation by Ad5-Infected A20 Cells

Splenocytes harvested from SPZ+CQ or RAS-immunized animals were stimulated by co-culture with infected/irradiated A20 cells in

96-well plates. Spleens were gently crushed using the flat end of a 3-cc or 10-cc syringe plunger, and the cell suspension was passed through a 70-μm filter. The splenocytes were washed with ice-cold 2% FBS/10 mM HEPES/1× Hank's balanced salt solution (HBSS), resuspended in RPMI, counted, diluted to 1×10^7 cells/mL in RPMI medium, and plated at 1×10^6 cells/well in 96-well plates. At 24 hr after infection, A20 cells were irradiated in a Pantak X-Rad 320 irradiator at 16,666 rads. After irradiation, the viable cell concentration was adjusted to 1.5×10^6 cells per mL and 1.5×10^5 infected cells were transferred to each well of U-bottom 96-well plates preloaded with 1×10^6 splenocytes from vaccinated or naive mice, in triplicate, and incubated for 5 hr at 37°C. BD Golgi Plug (BD Bioscience, San Jose, CA) was added 1 hr into the incubation to block cytokine release. Cells were centrifuged at 1,200 rpm for 5 min, the supernatant was flicked, and the cell pellets were resuspended by gentle vortexing. Live and dead cells were first stained with LIVE/DEAD Fixable Aqua stain kit (BD Biosciences, San Diego, CA), then the cells were blocked with FC Block (BD Biosciences, San Diego, CA). After blocking, cell surface markers were stained with the following antibodies (fluorochromes): CD4-eFluor-450 (clone RM4-5; eBioscience, San Diego, CA) and CD8a-PerCP-Cy5.5 (clone 53-6.7; BD Biosciences, San Diego, CA). Following fixation/permeabilization steps, the samples were stained intracellularly with the following antibodies (fluorochromes): IFN-γ-PE (clone XMG 1.2), TNF-α APC (clone MP6 XT22), and IL-2-Alexa488 (clone JES6-5H4) (BD Biosciences, San Diego, CA). The frequency of $CD4^+$ and $CD8^+$ T cells, as well as antigen-specific IFNγ, TNF-α, and IL-2 intracellular cytokine-positive T cells was determined in an 8-color upgraded FACSCalibur (Becton Dickinson Immunocytometry Systems, San Jose, CA) with a 96-well Automated Micro-sampling System (AMS) (Cytek, Fremont, CA). Data were analyzed using FloJo software (Treestar, Inc., Ashland, OR).

ICS Flow Cytometry Analysis

Stimulation by PF3D7_0932900 Peptide Pools

Splenocytes from GC46.PF3D7_0932900 immunized mice were harvest and plated at 2×10^6 cells per well in a 96-well v-bottom plate. Cells were stimulated for 4 hr in the presence of 20 μg/mL brefeldin A (Sigma-Aldrich, St. Louis, MO) with either 15-mer peptides for the PF3D7_0932900 antigen at 2 μg/mL, overlapping by 10 amino acids (Mimotopes, Victoria, Australia), or 1% DMSO as a negative control. Subsequently, cells were stained with the LIVE/DEAD Fixable Blue Dead Cell Stain Kit, for UV excitation (Invitrogen, Grand Island, NY), surface stained with CD14 phycoerythrin (PE) (clone Sa14-2; Life Technologies, Grand Island, NY), CD19 Brilliant Violet 650 (clone 6D5; Biolegend, San Diego, CA), CD3 Alexa 700 (clone 17A2; Biolegend, San Diego, CA), CCR7 PerCPCy5.5 (clone 4B12; eBioscience, San Diego, CA), CD44 Pacific Blue (clone IM7; Biolegend, San Diego, CA), and CD62L Brilliant Violet 786 (clone MEL-14; BD Biosciences, San Diego, CA) and permeabilized using Cytofix/Cytoperm reagent (BD Biosciences, San Diego, CA). Cells were then intracellularly stained with CD4 Brilliant Violet 605 (clone RM4-5; Biolegend, San Diego, CA), together with CD8 Horizon V500 (clone 53-6.7), TNF Cy7PE (clone MP6-XT22), IFNγ allophycocyanin (clone XMG1.2), and IL-2 FITC (clone JES6-5H4; BD Biosciences, San Diego, CA). Samples were acquired using an LSR Fortessa (Becton Dickinson Immunocytometry Systems, San Jose, CA) and data were analyzed using FlowJo version 9.4.11 (TreeStar Inc., Ashland, OR). To identify antigen-specific responses, cells were gated on forward scatter (threshold), exclusion of aggregates, and subsequently to include singlets, viable cells, $CD14^-$, $CD3^+$, $CD19^-$, $CD3^+$, lymphocytes, and either $CD4^+$ or $CD8^+$ populations.

ELISpot Assay

RAS splenocytes were stimulated in vitro with Ad5-transduced or PyCSP peptide-pulsed APCs and the number of *P. yoelii* antigen-specific IFNγ-secreting spot-forming cells was evaluated after a 36-hr culture period. Splenocytes were tested at 400,000, 200,000, and 100,000 per well, while APCs were tested at 100,000 per well. Assays were performed in triplicate and the number of IFNγ-secreting cells, recognized as spot-forming cells (SFCs), was counted using an automated ELISpot Reader manufactured by AutoImmun Diagnostika (AID) GmbH (Strassberg, Germany). Data were presented as the number of IFNγ-secreting SFCs per million spleen cells.

SUPPLEMENTAL INFORMATION

Supplemental Information includes two tables and can be found with this article online at http://dx.doi.org/10.1016/j.omtm.2017.08.003.

AUTHOR CONTRIBUTIONS

Conceptualization, J.T.B. and D.E.B.; Methodology, P.C. and M.S.; Formal Analysis, P.C., C.A.L., and J.T.B.; Investigation, P.C., C.A.L., G.E., B.A.M., J.B., and E.C.S; Writing – Original Draft, J.T.B.; Writing – Review & Editing, J.T.B., J.C.A, D.L.D., and D.E.B.; Visualization, J.T.B., C.A.L., and E.C.S.; Supervision, J.T.B., D.E.B., D.L.D., and J.C.A.; Project Administration, C.R.K, T.L.R., and E.V.; Funding Acquisition, J.T.B., C.R.K., and D.L.D.

CONFLICTS OF INTEREST

D.E.B. is employed at GenVec, Inc. J.T.B., P.C., B.A.M., G.E., and C.A.L. were employed at GenVec, Inc. at the time this work was performed.

ACKNOWLEDGMENTS

We thank Randy Osborn, Holly Torano, Damodar Ettyreddy, Elena Semenova, A'drian Pineda, Esteban Abot, Arnel Belmonte, Dianne Litilit, Rebecca Danner, Nonenipha Rangel, and Kyosuke Oda for their excellent technical assistance. We thank Rena Cohen for copy editing and assistance with submission of the manuscript. The views expressed in this article are those of the authors and do not necessarily reflect the official policy or position of the Department of the Navy, Department of Defense, nor the U.S. Government. M.S. and E.V. are employees of the U.S. Government. T.L.R. was a U.S. military officer at the time this work was performed. This work was prepared as part of their official duties. Title 17 U.S.C. §105 provides that "Copyright protection under this title is not available for any work of the United States Government." Title 17 U.S.C. §101 defines U.S. Government work as work prepared by a military service member

or employee of the U.S. Government as part of that person's official duties. P.C., G.E., B.A.M., C.R.K., D.E.B., and J.T.B. are/were employees of GenVec Inc., a for-profit corporation. This work was supported by the NIH (grants 1R43AIO84269-01 and 1R43AI100467-01 to J.T.B.), the Department of Defense (grant A043-189-0667 to J.T.B.), and the NMRC Military Infectious Disease Research Program (work unit number 602236N.04427.B27.A0242).

REFERENCES

1. Davies, D.H., Liang, X., Hernandez, J.E., Randall, A., Hirst, S., Mu, Y., Romero, K.M., Nguyen, T.T., Kalantari-Dehaghi, M., Crotty, S., et al. (2005). Profiling the humoral immune response to infection by using proteome microarrays: high-throughput vaccine and diagnostic antigen discovery. Proc. Natl. Acad. Sci. USA *102*, 547–552.
2. Nabel, G.J. (2013). Designing tomorrow's vaccines. N. Engl. J. Med. *368*, 551–560.
3. Krzych, U., Zarling, S., and Pichugin, A. (2014). Memory T cells maintain protracted protection against malaria. Immunol. Lett. *161*, 189–195.
4. Tsuji, M. (2010). A retrospective evaluation of the role of T cells in the development of malaria vaccine. Exp. Parasitol. *126*, 421–425.
5. Overstreet, M.G., Cockburn, I.A., Chen, Y.C., and Zavala, F. (2008). Protective CD8 T cells against Plasmodium liver stages: immunobiology of an 'unnatural' immune response. Immunol. Rev. *225*, 272–283.
6. Kaufmann, S.H. (2011). Fact and fiction in tuberculosis vaccine research: 10 years later. Lancet Infect. Dis. *11*, 633–640.
7. Appay, V., Douek, D.C., and Price, D.A. (2008). CD8+ T cell efficacy in vaccination and disease. Nat. Med. *14*, 623–628.
8. World Health Organization (2016). World Malaria Report. http://www.who.int/malaria/publications/world-malaria-report-2016/report/en/.
9. Nussenzweig, R.S., Vanderberg, J., Most, H., and Orton, C. (1967). Protective immunity produced by the injection of X-irradiated sporozoites of plasmodium berghei. Nature *216*, 160–162.
10. Clyde, D.F., Most, H., McCarthy, V.C., and Vanderberg, J.P. (1973). Immunization of man against sporozite-induced falciparum malaria. Am. J. Med. Sci. *266*, 169–177.
11. Hoffman, S.L., Goh, L.M., Luke, T.C., Schneider, I., Le, T.P., Doolan, D.L., Sacci, J., de la Vega, P., Dowler, M., Paul, C., et al. (2002). Protection of humans against malaria by immunization with radiation-attenuated Plasmodium falciparum sporozoites. J. Infect. Dis. *185*, 1155–1164.
12. Gwadz, R.W., Cochrane, A.H., Nussenzweig, V., and Nussenzweig, R.S. (1979). Preliminary studies on vaccination of rhesus monkeys with irradiated sporozoites of Plasmodium knowlesi and characterization of surface antigens of these parasites. Bull. World Health Organ. *57* (*Suppl 1*), 165–173.
13. Schofield, L., Villaquiran, J., Ferreira, A., Schellekens, H., Nussenzweig, R., and Nussenzweig, V. (1987). Gamma interferon, CD8+ T cells and antibodies required for immunity to malaria sporozoites. Nature *330*, 664–666.
14. Weiss, W.R., Sedegah, M., Beaudoin, R.L., Miller, L.H., and Good, M.F. (1988). CD8+ T cells (cytotoxic/suppressors) are required for protection in mice immunized with malaria sporozoites. Proc. Natl. Acad. Sci. USA *85*, 573–576.
15. Hoffman, S.L., Isenbarger, D., Long, G.W., Sedegah, M., Szarfman, A., Waters, L., Hollingdale, M.R., van der Meide, P.H., Finbloom, D.S., and Ballou, W.R. (1989). Sporozoite vaccine induces genetically restricted T cell elimination of malaria from hepatocytes. Science *244*, 1078–1081.
16. Rodrigues, M.M., Cordey, A.S., Arreaza, G., Corradin, G., Romero, P., Maryanski, J.L., Nussenzweig, R.S., and Zavala, F. (1991). CD8+ cytolytic T cell clones derived against the Plasmodium yoelii circumsporozoite protein protect against malaria. Int. Immunol. *3*, 579–585.
17. Romero, P., Maryanski, J.L., Corradin, G., Nussenzweig, R.S., Nussenzweig, V., and Zavala, F. (1989). Cloned cytotoxic T cells recognize an epitope in the circumsporozoite protein and protect against malaria. Nature *341*, 323–326.
18. Weiss, W.R., and Jiang, C.G. (2012). Protective CD8+ T lymphocytes in primates immunized with malaria sporozoites. PLoS ONE *7*, e31247.
19. Menard, R., Tavares, J., Cockburn, I., Markus, M., Zavala, F., and Amino, R. (2013). Looking under the skin: the first steps in malarial infection and immunity. Nat Rev Microbiol. *11*, 701–712.
20. Kumar, K.A., Sano, G., Boscardin, S., Nussenzweig, R.S., Nussenzweig, M.C., Zavala, F., and Nussenzweig, V. (2006). The circumsporozoite protein is an immunodominant protective antigen in irradiated sporozoites. Nature *444*, 937–940.
21. Doolan, D.L., Southwood, S., Freilich, D.A., Sidney, J., Graber, N.L., Shatney, L., Bebris, L., Florens, L., Dobano, C., Witney, A.A., et al. (2003). Identification of Plasmodium falciparum antigens by antigenic analysis of genomic and proteomic data. Proc. Natl. Acad. Sci. USA *100*, 9952–9957.
22. Doolan, D.L., Hoffman, S.L., Southwood, S., Wentworth, P.A., Sidney, J., Chesnut, R.W., Keogh, E., Appella, E., Nutman, T.B., Lal, A.A., et al. (1997). Degenerate cytotoxic T cell epitopes from P. falciparum restricted by multiple HLA-A and HLA-B supertype alleles. Immunity *7*, 97–112.
23. Doolan, D.L., Southwood, S., Chesnut, R., Appella, E., Gomez, E., Richards, A., Higashimoto, Y.I., Maewal, A., Sidney, J., Gramzinski, R.A., et al. (2000). HLA-DR-promiscuous T cell epitopes from Plasmodium falciparum pre-erythrocytic-stage antigens restricted by multiple HLA class II alleles. J. Immunol. *165*, 1123–1137.
24. Yayon, A., and Ginsburg, H. (1983). Chloroquine inhibits the degradation of endocytic vesicles in human malaria parasites. Cell Biol. Int. Rep. *7*, 895.
25. Roestenberg, M., McCall, M., Hopman, J., Wiersma, J., Luty, A.J., van Gemert, G.J., van de Vegte-Bolmer, M., van Schaijk, B., Teelen, K., Arens, T., et al. (2009). Protection against a malaria challenge by sporozoite inoculation. N. Engl. J. Med. *361*, 468–477.
26. Belnoue, E., Costa, F.T., Frankenberg, T., Vigário, A.M., Voza, T., Leroy, N., Rodrigues, M.M., Landau, I., Snounou, G., and Rénia, L. (2004). Protective T cell immunity against malaria liver stage after vaccination with live sporozoites under chloroquine treatment. J. Immunol. *172*, 2487–2495.
27. Mauduit, M., Tewari, R., Depinay, N., Kayibanda, M., Lallemand, E., Chavatte, J.M., Snounou, G., Rénia, L., and Grüner, A.C. (2010). Minimal role for the circumsporozoite protein in the induction of sterile immunity by vaccination with live rodent malaria sporozoites. Infect. Immun. *78*, 2182–2188.
28. Speake, C., and Duffy, P.E. (2009). Antigens for pre-erythrocytic malaria vaccines: building on success. Parasite Immunol. *31*, 539–546.
29. Tarun, A.S., Peng, X., Dumpit, R.F., Ogata, Y., Silva-Rivera, H., Camargo, N., Daly, T.M., Bergman, L.W., and Kappe, S.H. (2008). A combined transcriptome and proteome survey of malaria parasite liver stages. Proc. Natl. Acad. Sci. USA *105*, 305–310.
30. Zhou, Y., Ramachandran, V., Kumar, K.A., Westenberger, S., Refour, P., Zhou, B., Li, F., Young, J.A., Chen, K., Plouffe, D., et al. (2008). Evidence-based annotation of the malaria parasite's genome using comparative expression profiling. PLoS ONE *3*, e1570.
31. Williams, C.T., and Azad, A.F. (2010). Transcriptional analysis of the pre-erythrocytic stages of the rodent malaria parasite, Plasmodium yoelii. PLoS ONE *5*, e10267.
32. Bijker, E.M., Bastiaens, G.J., Teirlinck, A.C., van Gemert, G.J., Graumans, W., van de Vegte-Bolmer, M., Siebelink-Stoter, R., Arens, T., Teelen, K., Nahrendorf, W., et al. (2013). Protection against malaria after immunization by chloroquine prophylaxis and sporozoites is mediated by preerythrocytic immunity. Proc Natl Acad Sci U S A *110*, 7862–7867.
33. Bruder, J.T., Semenova, E., Chen, P., Limbach, K., Patterson, N.B., Stefaniak, M.E., Konovalova, S., Thomas, C., Hamilton, M., King, C.R., et al. (2012). Modification of Ad5 hexon hypervariable regions circumvents pre-existing Ad5 neutralizing antibodies and induces protective immune responses. PLoS ONE *7*, e33920.
34. McVey, D., Cranfield, M.R., Torano, H., Kuete, H.M., Lee, G.P., Glenn, A., Bruder, J.T., Rangel, D., Brough, D.E., and Gall, J.G. (2013). Adenoviruses isolated from wild gorillas are closely related to human species C viruses. Virology *444*, 119–123.
35. Johnson, T.R., Rangel, D., Graham, B.S., Brough, D.E., and Gall, J.G. (2014). Genetic vaccine for respiratory syncytial virus provides protection without disease potentiation. Mol. Ther. *22*, 196–205.
36. Michiels, F., van Es, H., van Rompaey, L., Merchiers, P., Francken, B., Pittois, K., van der Schueren, J., Brys, R., Vandersmissen, J., Beirinckx, F., et al. (2002). Arrayed

adenoviral expression libraries for functional screening. Nat. Biotechnol. *20*, 1154–1157.

37. Arthur, J.F., Butterfield, L.H., Roth, M.D., Bui, L.A., Kiertscher, S.M., Lau, R., Dubinett, S., Glaspy, J., McBride, W.H., and Economou, J.S. (1997). A comparison of gene transfer methods in human dendritic cells. Cancer Gene Ther. *4*, 17–25.

38. Mishra, S., Rai, U., Shiratsuchi, T., Li, X., Vanloubbeeck, Y., Cohen, J., Nussenzweig, R.S., Winzeler, E.A., Tsuji, M., and Nussenzweig, V. (2011). Identification of non-CSP antigens bearing CD8 epitopes in mice immunized with irradiated sporozoites. Vaccine *29*, 7335–7342.

39. Hafalla, J.C., Bauza, K., Friesen, J., Gonzalez-Aseguinolaza, G., Hill, A.V., and Matuschewski, K. (2013). Identification of targets of $CD8^{+}$ T cell responses to malaria liver stages by genome-wide epitope profiling. PLoS Pathog. *9*, e1003303.

40. Chuang, I., Sedegah, M., Cicatelli, S., Spring, M., Polhemus, M., Tamminga, C., Patterson, N., Guerrero, M., Bennett, J.W., McGrath, S., et al. (2013). DNA prime/adenovirus boost malaria vaccine encoding P. falciparum CSP and AMA1 induces sterile protection associated with cell-mediated immunity. PLoS ONE *8*, e55571.

41. Aguiar, J.C., Bolton, J., Wanga, J., Sacci, J.B., Iriko, H., Mazeika, J.K., Han, E.T., Limbach, K., Patterson, N.B., Sedegah, M., et al. (2015). Discovery of novel Plasmodium falciparum pre-erythrocytic antigens for vaccine development. PLoS ONE *10*, e0136109.

42. Chaves, F.A., Lee, A.H., Nayak, J.L., Richards, K.A., and Sant, A.J. (2012). The utility and limitations of current Web-available algorithms to predict peptides recognized by CD4 T cells in response to pathogen infection. J. Immunol. *188*, 4235–4248.

43. Fleri, W., Paul, S., Dhanda, S.K., Mahajan, S., Xu, X., Peters, B., and Sette, A. (2017). The Immune Epitope Database and Analysis Resource in epitope discovery and synthetic vaccine design. Front. Immunol. *8*, 278.

44. Butler, N.S., Schmidt, N.W., Vaughan, A.M., Aly, A.S., Kappe, S.H., and Harty, J.T. (2011). Superior antimalarial immunity after vaccination with late liver stage-arresting genetically attenuated parasites. Cell Host Microbe *9*, 451–462.

45. Doll, K.L., Butler, N.S., and Harty, J.T. (2014). CD8 T cell independent immunity after single dose infection-treatment-vaccination (ITV) against Plasmodium yoelii. Vaccine *32*, 483–491.

46. World Health Organization (2014). WHO Preferred Product Characteristics (PPC) for Malaria Vaccines. http://www.euvaccine.eu/sites/default/files/uploads/Files/3-WHO_IVB_14.09_eng.pdf.

47. Limbach, K., Stefaniak, M., Chen, P., Patterson, N.B., Liao, G., Weng, S., Krepkiy, S., Ekberg, G., Torano, H., Ettyreddy, D., et al. (2017). New gorilla adenovirus vaccine vectors induce potent immune responses and protection in a mouse malaria model. Malar. J. *16*, 263.

48. Quinn, K.M., Da Costa, A., Yamamoto, A., Berry, D., Lindsay, R.W., Darrah, P.A., Wang, L., Cheng, C., Kong, W.P., Gall, J.G., et al. (2013). Comparative analysis of the magnitude, quality, phenotype, and protective capacity of simian immunodeficiency virus gag-specific CD8+ T cells following human-, simian-, and chimpanzee-derived recombinant adenoviral vector immunization. J. Immunol. *190*, 2720–2735.

49. Barnes, E., Folgori, A., Capone, S., Swadling, L., Aston, S., Kurioka, A., Meyer, J., Huddart, R., Smith, K., Townsend, R., et al. (2012). Novel adenovirus-based vaccines induce broad and sustained T cell responses to HCV in man. Sci. Transl. Med. *4*, 115ra1.

50. Ewer, K.J., O'Hara, G.A., Duncan, C.J., Collins, K.A., Sheehy, S.H., Reyes-Sandoval, A., Goodman, A.L., Edwards, N.J., Elias, S.C., Halstead, F.D., et al. (2013). Protective CD8+ T-cell immunity to human malaria induced by chimpanzee adenovirus-MVA immunisation. Nat. Commun. *4*, 2836.

51. Ledgerwood, J.E., Sullivan, N.J., and Graham, B.S. (2015). Chimpanzee adenovirus vector Ebola vaccine–preliminary report. N. Engl. J. Med. *373*, 776.

52. Ogwang, C., Kimani, D., Edwards, N.J., Roberts, R., Mwacharo, J., Bowyer, G., Bliss, C., Hodgson, S.H., Njuguna, P., Viebig, N.K., et al.; MVVC group (2015). Prime-boost vaccination with chimpanzee adenovirus and modified vaccinia Ankara encoding TRAP provides partial protection against Plasmodium falciparum infection in Kenyan adults. Sci. Transl. Med. *7*, 286re5.

53. Ewer, K., Rampling, T., Venkatraman, N., Bowyer, G., Wright, D., Lambe, T., Imoukhuede, E.B., Payne, R., Fehling, S.K., Strecker, T., et al. (2015). A monovalent chimpanzee adenovirus ebola vaccine - preliminary report. N. Engl. J. Med. *374*, 1635–1646.

54. Tapia, M.D., Sow, S.O., Lyke, K.E., Haidara, F.C., Diallo, F., Doumbia, M., Traore, A., Coulibaly, F., Kodio, M., Onwuchekwa, U., et al. (2016). Use of ChAd3-EBO-Z Ebola virus vaccine in Malian and US adults, and boosting of Malian adults with MVA-BN-Filo: a phase 1, single-blind, randomised trial, a phase 1b, open-label and double-blind, dose-escalation trial, and a nested, randomised, double-blind, placebo-controlled trial. Lancet Infect. Dis. *16*, 31–42.

55. Limbach, K., Aguiar, J., Gowda, K., Patterson, N., Abot, E., Sedegah, M., Sacci, J., and Richie, T. (2011). Identification of two new protective pre-erythrocytic malaria vaccine antigen candidates. Malar. J. *10*, 65.

56. Doolan, D.L., Sedegah, M., Hedstrom, R.C., Hobart, P., Charoenvit, Y., and Hoffman, S.L. (1996). Circumventing genetic restriction of protection against malaria with multigene DNA immunization: CD8+ cell-, interferon gamma-, and nitric oxide-dependent immunity. J. Exp. Med. *183*, 1739–1746.

57. Aurrecoechea, C., Brestelli, J., Brunk, B.P., Dommer, J., Fischer, S., Gajria, B., Gao, X., Gingle, A., Grant, G., Harb, O.S., et al. (2009). PlasmoDB: a functional genomic database for malaria parasites. Nucleic Acids Res. *37*, D539–D543.

58. Gibson, D.G., Young, L., Chuang, R.Y., Venter, J.C., Hutchison, C.A., 3rd, and Smith, H.O. (2009). Enzymatic assembly of DNA molecules up to several hundred kilobases. Nat. Methods *6*, 343–345.

59. Katzen, F. (2007). Gateway(®) recombinational cloning: a biological operating system. Expert Opin. Drug Discov. *2*, 571–589.

60. Bruder, J.T., Chen, P., Semenova, E., Thomas, C.A., Konovalova, S., Ekberg, G., Ettyreddy, D., McVey, D., Gall, J.G., King, C.R., and Brough, D.E. (2013). Identification of a suppressor mutation that improves the yields of hexon-modified adenovirus vectors. J. Virol. *87*, 9661–9671.

61. Kappler, J., White, J., Wegmann, D., Mustain, E., and Marrack, P. (1982). Antigen presentation by Ia+ B cell hybridomas to H-2-restricted T cell hybridomas. Proc. Natl. Acad. Sci. USA *79*, 3604–3607.

62. Sedegah, M., Rogers, W.O., Belmonte, A., Belmonte, M., Banania, G., Patterson, N., Ferrari, M., Kaslow, D.C., Carucci, D.J., Richie, T.L., and Doolan, D.L. (2006). Vaxfectin enhances immunogenicity and protective efficacy of P. yoelii circumsporozoite DNA vaccines. Vaccine *24*, 1921–1927.

63. Doolan, D.L., and Hoffman, S.L. (1999). IL-12 and NK cells are required for antigen-specific adaptive immunity against malaria initiated by CD8+ T cells in the Plasmodium yoelii model. J. Immunol. *163*, 884–892.

OMTM, Volume 7

Supplemental Information

Profiling the Targets of Protective CD8$^+$ T Cell Responses to Infection

Joseph T. Bruder, Ping Chen, Greg Ekberg, Emily C. Smith, Christopher A. Lazarski, Bennett A. Myers, Jessica Bolton, Martha Sedegah, Eileen Villasante, Thomas L. Richie, C. Richter King, Joao C. Aguiar, Denise L. Doolan, and Douglas E. Brough

Supplemental Information:

Table S1. Vector yields on various size plates

Plate Size	Adg.*Py*HEP17		Adg.CMVp65	
	ffu/ml	ffu total	ffu/ml	ffu total
60 mm	3.10E+08	1.30E+09	8.20E+08	3.44E+09
6 well	4.55E+08	9.55E+08	6.70E+08	1.41E+09
12 well	9.00E+08	7.56E+08	1.10E+09	9.24E+08
24 well	1.33E+09	5.59E+08	1.02E+09	4.28E+08
48 well	1.38E+09	5.80E+07	1.27E+09	5.30E+07
96 well	1.26E+09	2.26E+07	1.19E+09	2.10E+07

Table S2. Thirteen antigens that recalled $CD8^+$ T cell responses more frequent than CSP from mice immunized with *P. yoelii* SPZ+CQ

Rank	Gene ID *P. yoelii*	Gene ID *P. falciparum*	% Identity [a]	*P. yoelii* Protein Name - Function	Liver stage active genes from Tarum et al., 2008	
					Transcriptome Clusters [h]	Proteome
1	PY02605	PF3D7_0932900	40	Hypothetical Protein	C3	-
2	PY02989	PF3D7_0212800	47	Hypothetical Protein [b]	C4	-
3	PY02326	PF3D7_1309500	74	snornp protein gar1 [c]	C3	+
4	PY02030	PF3D7_0624000	86	hexokinase		+
5	PY00629	PF3D7_0108300	33	asparagine-rich protein, putative		+
6	PY07062	PF3D7_1446400	82	pyruvate dehydrogenase E1 beta subunit	C3	+
7	PY00361	PF3D7_1357100	99	translation elongation factor EF-1, subunit alpha		+
8	PY04421	PF3D7_1121600	56	U43539 hepatocyte erythrocyte protein 17 kDa [d]	C3	+
9	PY05048	PF3D7_1108400	95	Protein kinase domain [e]		+
10	PY07825	PF3D7_0814200	86	Arabidopsis thaliana At1g20220/T20H2_3-related [f]	C3	+
11	PY05748	PF3D7_0930300	32	merozoite surface protein 1 precursor	C1	-
12	PY04475	PF3D7_0621200	86	ethylene-inducible protein hever [g]		+
13	PY02148	PF3D7_1334600	31	merozoite surface protein 7 precursor	C1	-

[a] Percent identity between *P. falciparum* and *P. yoelii* orthologues established using blastpp suite-2sequences, National Center for Biotechnology Information.

[b] *P. falciparum* protein function is multidrug efflux pump, putative.

[c] *P. falciparum* protein function is H/ACA ribonucleoprotein complex subunit 1, putative.

[d] *P. falciparum* protein function is exported protein 1.

[e] *P. falciparum* protein function is casein kinase 2, alpha subunit.

[f] *P. falciparum* protein function is DNA/RNA-binding protein Alba 1.

[g] *P. falciparum* protein function pyridoxine biosynthesis protein PDX1.

[h] Clusters from Tarum *et al*., 2008 - C1 includes LS genes mainly up-regulated in LS24 compared with LS40 and LS50, but that were also expressed in the mosquito and erythrocytic stages.C2 encompassed genes that were mainly up-regulated at LS24; C3 showed most of the genes up-regulated in all three LS time points queried. C4 showed genes mainly up-regulated in LS40 and LS50.
